# Characterization of minority HIV-1 drug resistant variants in the United Kingdom following the verification of a deep sequencing-based HIV-1 genotyping and tropism assay

**DOI:** 10.1186/s12981-018-0206-y

**Published:** 2018-11-08

**Authors:** Nicholas Silver, Mary Paynter, Georgina McAllister, Maureen Atchley, Christine Sayir, John Short, Dane Winner, David J. Alouani, Freddie H. Sharkey, Kicki Bergefall, Kate Templeton, David Carrington, Miguel E. Quiñones-Mateu

**Affiliations:** 1 St. George’s University Hospitals NHS Foundation Trust, London, UK; 2 NHS Lothian, Edinburgh, UK; 3 University Hospital Translational Laboratory, University Hospitals Cleveland Medical Center, Cleveland, OH USA; 4 ThermoFisher Scientific, Paisley, UK; 5 Departments of Pathology, Case Western Reserve University, 2109 Adelbert Rd., Wood Bldg., Room W210C, Cleveland, OH 44106-4960 USA; 6 Departments of Medicine, Case Western Reserve University, Cleveland, OH USA

**Keywords:** HIV-1, Drug resistance, Minority variants, Deep sequencing, United Kingdom

## Abstract

**Background:**

The widespread global access to antiretroviral drugs has led to considerable reductions in morbidity and mortality but, unfortunately, the risk of virologic failure increases with the emergence, and potential transmission, of drug resistant viruses. Detecting and quantifying HIV-1 drug resistance has therefore become the standard of care when designing new antiretroviral regimens. The sensitivity of Sanger sequencing-based HIV-1 genotypic assays is limited by its inability to identify minority members of the quasispecies, i.e., it only detects variants present above ~ 20% of the viral population, thus, failing to detect minority variants below this threshold. It is clear that deep sequencing-based HIV-1 genotyping assays are an important step change towards accurately monitoring HIV-infected individuals.

**Methods:**

We implemented and verified a clinically validated HIV-1 genotyping assay based on deep sequencing (DEEPGEN™) in two clinical laboratories in the United Kingdom: St. George’s University Hospitals Healthcare NHS Foundation Trust (London) and at NHS Lothian (Edinburgh), to characterize minority HIV-1 variants in 109 plasma samples from ART-naïve or -experienced individuals.

**Results:**

Although subtype B HIV-1 strains were highly prevalent (44%, 48/109), most individuals were infected with non-B subtype viruses (i.e., A1, A2, C, D, F1, G, CRF02_AG, and CRF01_AE). DEEPGEN™ was able to accurately detect drug resistance-associated mutations not identified using standard Sanger sequencing-based tests, which correlated significantly with patient’s antiretroviral treatment histories. A higher proportion of minority PI-, NRTI-, and NNRTI-resistance mutations was detected in NHS Lothian patients compared to individuals from St. George’s, mainly M46I/L and I50 V (associated with PIs), D67 N, K65R, L74I, M184 V/I, and K219Q (NRTIs), and L100I (NNRTIs). Interestingly, we observed an inverse correlation between intra-patient HIV-1 diversity and CD4^+^ T cell counts in the NHS Lothian patients.

**Conclusions:**

This is the first study evaluating the transition, training, and implementation of DEEPGEN™ between three clinical laboratories in two different countries. More importantly, we were able to characterize the HIV-1 drug resistance profile (including minority variants), coreceptor tropism, subtyping, and intra-patient viral diversity in patients from the United Kingdom, providing a rigorous foundation for basing clinical decisions on highly sensitive and cost-effective deep sequencing-based HIV-1 genotyping assays in the country.

**Electronic supplementary material:**

The online version of this article (10.1186/s12981-018-0206-y) contains supplementary material, which is available to authorized users.

## Background

The United Kingdom (U.K.) has a relatively small HIV-1 epidemic, with just over 100,000 people living with HIV-1 and an adult prevalence of 0.16%, despite the recent increase in the annual number of new diagnoses, particularly in people born in the country [1]. Nonetheless, the U.K. is a clear example of how access to combination antiretroviral therapy (cART) can transform a national HIV-1 epidemic: 98% of the people living with HIV-1 were receiving cART in 2017 with 97% achieving virus suppression [1, 2]. The prevalence of resistance to any antiretroviral drug among ART-experienced patients in the country seems to have remained stable -around 30%- since 2011, while transmitted HIV-1 drug resistance (prevalence in ART-naïve individuals) is approximately 7% [3–6]. These results highlight the fact that monitoring HIV-1 drug resistance is not only crucial to controlling plasma viremia in patients receiving antiretroviral drugs but also in the surveillance of transmitted drug resistance, a critical public health issue in the fight against HIV/AIDS.

HIV-1 genotyping assays, based on population (Sanger) sequencing, have been the most common method to manage patients infected with HIV-1 for almost 20 years [7–11]. Our current understanding of HIV-1 drug resistance, and the great success controlling HIV-1 disease during the last decade, have been the result of a myriad of HIV-1 studies using this standard methodology [7, 12, 13]. Nevertheless, HIV-1 genotypes based on Sanger sequencing can only detect HIV-1 variants present at frequencies above approximately 20% of the viral quasispecies [14–18], failing to quantify low-levels of HIV-1 drug resistant variants [10, 19]. These variants, usually present as minority members of the virus population, can be selected and become predominant under the appropriate pressure by antiretroviral drugs [20–22]. With the advent of deep (next-generation) sequencing, several new HIV-1 genotyping approaches based on this ultrasensitive methodology have been developed with the goal of detecting drug resistant HIV-1 variants at low frequencies, i.e., below 20% of the viral population [19, 23–26], with only a few assays being used in the clinical setting [19, 27, 28]. Although the clinical significance of these minority drug resistant HIV-1 variants is still on discussion [29–33], numerous groups are now using these assays not only to monitor HIV-1 drug resistance but also to better understand the role of low-level HIV-1 variants on transmission, disease progression, and HIV-1 cure strategies [reviewed on [10, 11]].

Several groups in the U.K. have used deep sequencing to investigate minority HIV-1 variants associated with transmitted drug resistance [3, 6, 34], selection and prevalence of low-abundance drug resistant HIV-1 variants [35], genetic diversity in full-length HIV-1 genomes [36], HIV-1 coreceptor tropism [37–39], and their potential contribution to virologic failure [40]; however, in-house HIV-1 genotyping based on deep sequencing is only available in reference laboratories in the United Kingdom. In this verification study, we implemented DEEPGEN™, a validated deep sequencing-based HIV-1 genotyping assay used in a CLIA/CAP-accredited laboratory in the United States since 2013 [19] and in Uganda since January 2017 [41], in two clinical laboratories in the U.K. i.e., St. George’s University Hospitals Healthcare NHS Foundation Trust (London) and at NHS Lothian (Edinburgh). A comprehensive list of comparative studies first verified the feasibility of using DEEPGEN™ to monitor HIV-infected individuals in the U.K., while we characterized majority and minority drug resistant HIV-1 variants in these cohorts of patients and their correlation with virological and immunological parameters.

## Methods

### Clinical samples

A total of 109 convenience plasma samples were obtained during routine patient monitoring from two well-characterized cohorts of HIV-infected individuals at St. George’s University Hospitals Healthcare NHS Foundation Trust (London, United Kingdom) and at NHS Lothian (Edinburgh, United Kingdom), with the written informed consent of each participant as described in local clinical research protocols. Clinical, virological, and demographics data, including HIV-1 drug resistance results based on standard Sanger sequencing performed locally and/or in reference laboratories testing, were obtained from patient care databases at the respective hospitals (Additional file 1: Table S1, Additional file 2: Table S2).

### HIV-1 genotyping and tropism determination based on deep sequencing of the *gag*-p2/NCp7/p1/p6/*pol*-PR/RT/IN- and *env*-C2V3-coding regions

HIV-1 drug resistance and co-receptor tropism was determined using an all-inclusive deep sequencing-based assay, DEEPGEN™, as described [19]. Briefly, plasma viral RNA was purified and three RT-PCR products corresponding to the *gag*-p2/NCp7/p1/p6/*pol*-PR/RT- (1657 bp fragment), *pol*-IN- (1114 bp fragment), and *env*-C2V3- (480 bp fragment) coding region of HIV-1 amplified. These amplicons were purified, quantified, and used to construct a multiplexed library for shotgun sequencing on the Ion Personal Genome Machine (PGM, ThermoFisher Scientific). Reads were mapped and aligned against sample-specific reference sequences constructed for the *gag*-p2/NCp7/p1/p6/*pol*-PR/RT/IN or *env*-gp120 HIV-1 genomic regions using the DEEPGEN™ Software Tool Suite v2 (Alouani and Quiñones-Mateu, unpublished) as described [19]. Plasma samples were classified as containing non-R5 viruses if at least 2% of the individual sequences, as determined by deep sequencing, were predicted to be non-R5 [42, 43]. In this study, minority variants were defined as amino acid substitutions detected at ≥ 1% (based on the intrinsic error rate of the system [19]) and < 20% of the virus population, corresponding to those mutations that cannot be determined using population sequencing [14–18].

### Phylogenetic and HIV-1 diversity analysis

Three consensus sequences, corresponding to the three amplicons (PR/RT, INT, and C2V3), were generated for each patient-derived virus, aligned using ClustalW [44] and their phylogeny reconstructed using the neighbor-joining statistical method as implemented within MEGA 6.06 [45]. HIV-1 subtype, initially predicted by phylogenetic analysis, was confirmed using *pol*-PR/RT/INT and *env*-V3 sequences with the DEEPGEN™ Software Tool Suite v2 and Geno2Pheno tools (http://www.geno2pheno.org). Inter-patient genetic distances were determined using the Maximum Composite Likelihood model with bootstrap as the variance estimation method (1000 replicates) within MEGA 6.06 [45]. Intra-patient HIV-1 quasispecies diversity was determined using all three PR/RT-, INT-, and C2V3- coding regions based on the p-distance model as described for deep sequencing [46].

### Statistical analyses

Descriptive results are expressed as median values, standard deviations, range, and confidence intervals. The non-parametric Kruskal–Wallis one-way analysis of variance test was used to compare the mutations detected among the different groups. All differences with a *p* value of < 0.05 were considered statistically significant. The kappa coefficient, calculated using ComKappa2 v.2.0.4 [47], was used to quantify the concordance between HIV-1 coreceptor tropism determinations. The kappa coefficient calculates a chance-adjusted measure of the agreement between any number of categories, in this case HIV-1 coreceptor tropism determined by the same assay in two different locations. All statistical analyses were performed using GraphPad Prism v.6.0b (GraphPad Software, La Jolla, CA) unless otherwise specified. *gag*-p2/NCp7/p1/p6/*pol*-PR/RT/IN and *env*-C2V3 nucleotide sequences obtained by deep sequencing in this study have been submitted to the Los Alamos National Laboratory HIV-db Next Generation Sequence Archive (http://www.hiv.lanl.gov/content/sequence/HIV/NextGenArchive/Silver2018).

## Results

### Epidemiological, clinical, and virological characteristics of HIV-infected individuals

As described above, for this study we selected 109 plasma samples from HIV-1 patients being monitored at two hospitals in the United Kingdom: 59 from St. George’s University Hospitals Healthcare NHS Foundation Trust (St. George’s) and 50 from NHS Lothian (Table 1 and Additional file 1: Table S1, Additional file 2: Table S2). Participants from both institutions had similar median ages, i.e., 44 years (interquartile range, IQR: 35–51) and 42 years (IQR: 27–47) for St. George’s and NHS Lothian, respectively. Male gender was more frequent in the cohort of patients from NHS Lothian (68%) than in St. George’s (47%). Although not significant, median CD4^+^ T-cell counts were slightly lower in NHS Lothian patients (290 cells/mm^3^, IQR: 106-485) than in St. George’s patients (340 cells/mm^3^, IQR: 157–490), corresponding in both cases to relatively high median plasma HIV-1 RNA loads (4.62 log_10_, IQR: 3.81–5.15 and 4.76 log_10_, IQR: 4.36–5.19 for St. George’s and NHS Lothian patients, respectively). Most of the HIV-infected individuals from St. George’s were antiretroviral-experienced (45/59, 76%), while 56% (28/50) of NHS Lothian patients were antiretroviral-naïve. At the time of the study, most patients on treatment had received a median of 2.3 PIs (IQR: 0-5), all were treated with NRTIs (median 2.9 and 3.6 NRTIs for St. George’s and NHS Lothian patients, respectively), approximately half of the patients in each cohort were exposed to NNRTIs (24/45 and 12/22), and around one-third to INSTIs (17/45 and 6/22). Only four individuals from St. George’s were treated with the entry inhibitor maraviroc (Table 1, details in Additional file 1: Table S1, Additional file 2: Table S2).

**Table 1:** Demographic, clinical and virological characteristics

Characteristic	St. George’s (n = 59)	NHS Lothian (n = 50)
Median age (IQR)^a^	44 (35–51)	42 (27–47)
No. males (%)^b^	28 (47)	34 (68)
Risk factor (#) heterosexual^c^	5	1
MSM	4	12
IVDU	0	1
MTCT	0	3
Not determined	50	33
Median HIV-1 RNA (IQR), log_10_ c/ml^d^	4.62 (3.81–5.15)	4.76 (4.36–5.19)
Median CD4^+^ T cells (IQR), cell/mm^3e^	340 (157–490)	290 (106–485)
cART history—no. patients (%) Naive^f^	14 (24)	28 (56)
Experienced	45 (76)	22 (44)
cART regimen—no. patients (mean, range number of drugs) PI^g^	35 (2.3, 0–5)	17 (2.3, 0–5)
NRTI	45 (2.9, 1–7)	22 (3.6, 2–6)
NNRTI	24 (0.4, 0–1)	12 (0.8, 0–2)
INSTI	17 (0.4, 0–1)	6 (0.3, 0–1)
EI	4 (0.09, 0–1)	None

PI, protease inhibitors; NRTI, nucleoside reverse transcriptase inhibitors; NNRTI, non-nucleoside reverse transcriptase inhibitors; INSTI, integrase strand transfer inhibitors; entry inhibitors

^a^Median age at the time of sampling; IQR, interquartile range

^b^Number of male patients

^c^Most likely mode of HIV-1 transmission: heterosexual; MSM, men who have sex with men; IVDU, intravenous drug user; MTCT, mother-to-child transmission; n.d., not determined

^d^Median HIV-1 RNA plasma load (log_10_ copies/ml) and IQR at the time the blood sample was obtained

^e^Median CD4^+^ T-cell count (cells/mm^3^) and IQR at the time the blood sample was obtained

^f^Number of patients treated (experienced) or not (naïve) with combination antiretroviral therapy (cART) at the time the blood sample was obtained

^g^Number of patients treated with cART, and mean number of antiretroviral drugs used per patient

### Implementing DEEPGEN™ in the United Kingdom

**Fig. 1:**
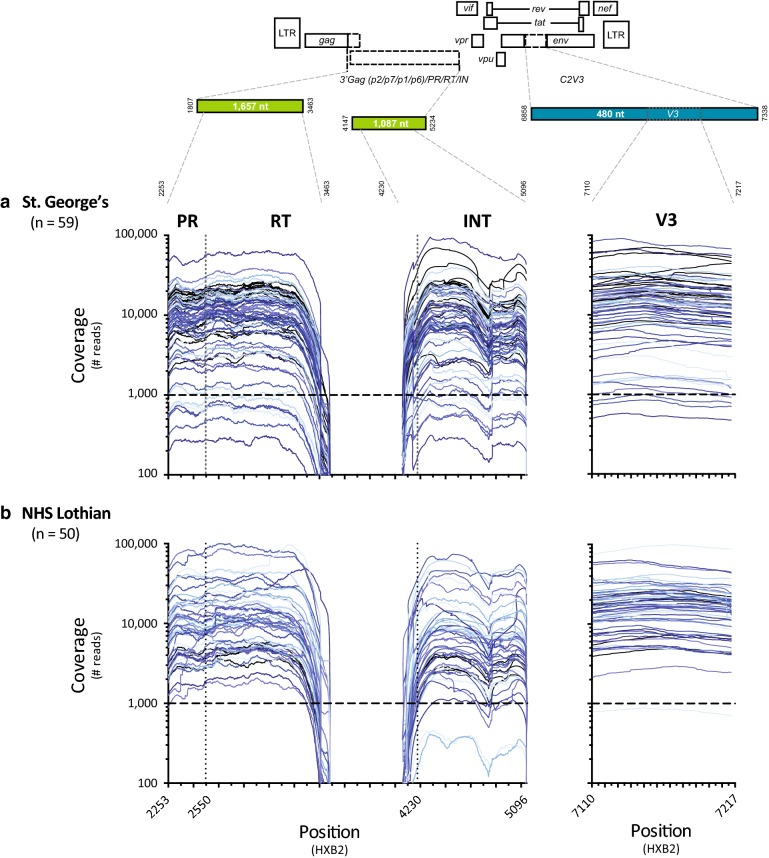
Number of mapped reads per nucleotide position (coverage) obtained by deep sequencing HIV-1 strains derived from 109 HIV-infected individuals, i.e., **a** 59 from St. George’s University Hospital Healthcare NHS Foundation Trust, London and **b** 50 from NHS Lothian, Edinburgh. The *pol*-PR/RT, *pol*-IN and *env*-C2V3 fragments from all 109 viruses were RT-PCR amplified and deep sequenced as described (19). Size of the amplicons and HIV-1 regions sequenced are indicated. See “Methods” for details

Our deep sequencing-based HIV-1 genotyping and coreceptor tropism assay (DEEPGEN™) has been characterized and validated for clinical use in the US. since late 2013 [19]. Here we transferred the technology to two independent clinical laboratories in the U.K. (St. George’s and NHS Lothian) to evaluate assay performance and the feasibility to detect minority HIV-1 drug resistance variants in different populations of HIV-infected individuals. Each clinical laboratory multiplexed their respective samples into three Ion 318 chips with median loading efficiencies of 65% and 61%, generating a total of 11,831,865 and 11,542,222 quality reads, with equal median read lengths of 226 bp for St. George’s and NHS Lothian, respectively. Although comparable, the average sequencing coverage at each nucleotide position varied with each sample and HIV-1 genomic region analyzed, with no significant differences between laboratories, i.e., PR/RT/INT (mean 7205 and 8878 reads) and V3 (16,893 and 20,218 reads) for St. George’s and NHS Lothian, respectively (Fig. 1). More importantly, these metrics ensured the minimum coverage of 1000 per nucleotide position sequenced required guaranteeing the detection of a minor variant present at least at 1% of the population [48].

**Fig. 2:**
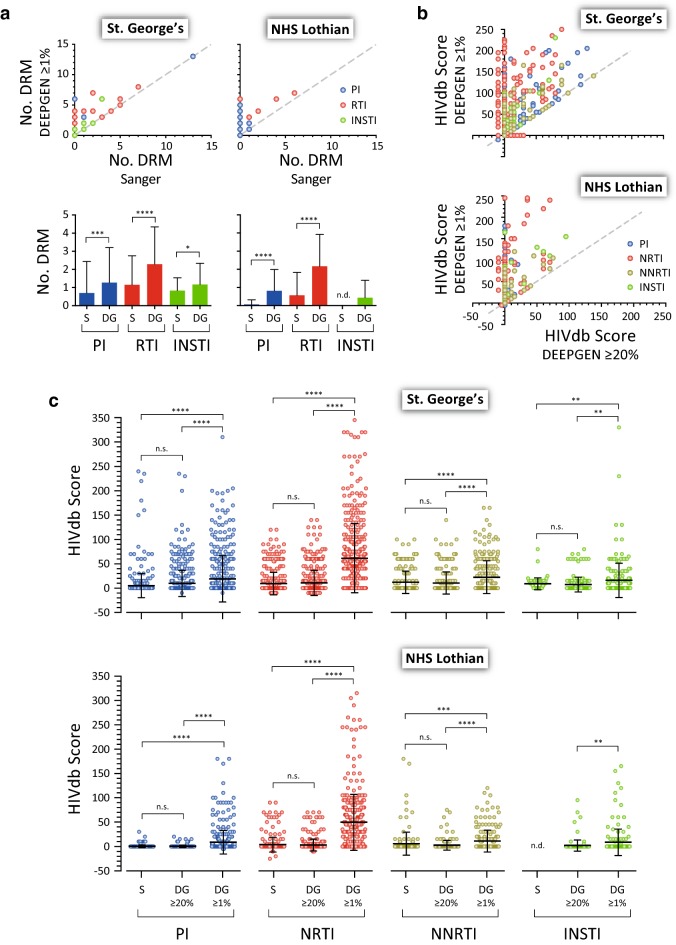
Comparing the determination of HIV-1 drug resistance using standard population (Sanger) and deep (DEEPGEN™) sequencing in two clinical laboratories in the U.K. Plasma samples from 59 (St. George’s) and 50 (NHS Lothian) treatment-experienced or -naive HIV-infected individuals were analyzed with Sanger sequencing and DEEPGEN™ as described in “Methods”. **a** Top plot compares the number of drug resistance mutations (DRM) detected by Sanger sequencing and DEEPGEN™ in each cohort of patients. Each dot corresponds to the number of mutations associated with resistance to protease (PI), reverse transcriptase (RTI) and integrase strand-transfer (INSTI) inhibitors in each patient. The mean difference in the numbers of drug resistance mutations detected by Sanger (S) and DEEPGEN™ (DG) associated with PIs, RTIs, and INSTIs in each cohort of patients is indicated in the bottom graph. **b** Comparing HIV-1 genotypic resistance interpretation using DEEPGEN™ with two different thresholds for mutation frequency: the standard ≥ 1% (19) and ≥ 20%, equivalent to the limit of detection for Sanger sequencing. A list of all the amino acid substitutions (mutations) identified for all 109 viruses with DEEPGEN™ at both thresholds was used with the HIVdb Program Genotypic Resistance Interpretation Algorithm from the Stanford University HIV Drug Resistance Database (http://hivdb.stanford.edu) to infer the levels of susceptibility to protease, reverse transcriptase, and integrase inhibitors (i.e., HIVdb scores). **c** Comparing the mean differences in the HIVdb scores determined using Sanger sequencing (S) or DEEPGEN™ (DG) at two thresholds (≥ 1% and ≥ 20%) by drug class: with PI, nucleoside/tide reverse transcriptase (NRTI), non-nucleoside reverse transcriptase (NNRTI), and INSTI inhibitors. Means ± standard deviations and statistically significant differences (unpaired t test) are marked by ****(*p *< 0.0001), ***(*p* < 0.001), **(*p* < 0.01), *(*p* < 0.05), and n.s. (*p* > 0.05). n.d., not determined

All 109 plasma samples from both cohorts of patients (St. George’s and NHS Lothian) were originally analyzed using standard Sanger-based HIV-1 genotyping in the respective clinical laboratories. Altogether a total of 157 mutations (129 and 28 in St. George’s and NHS Lothian cohorts, respectively) in positions associated with drug resistance were detected by Sanger sequencing (i.e., 44 in the protease, 93 in the RT, and 20 in the integrase) (Fig. 2a). As expected, all the drug resistance mutations identified by Sanger sequencing were also detected using DEEPGEN™, while 280 additional drug resistance mutations (120 and 160 in the St. George’s and NHS Lothian cohorts, respectively) were detected only by deep sequencing (i.e., 80 in the protease, 168 in the RT, and 32 in the integrase) (Fig. 2a). This difference in the numbers of drug resistance mutations detected by Sanger and deep sequencing—in both institutions—was significant, even when the mutations were quantified by drug class, ranging from 1.4- to 12-fold additional mutations detected by deep sequencing compared to Sanger sequencing (Paired *t* test, *p* < 0.0001 to *p* = 0.029) (Fig. 2a).

Overall, a similar number of drug resistance mutations were identified using Sanger sequencing and DEEPGEN™ with a mutation frequency threshold of ≥ 20%, resulting in comparable HIVdb mutation scores; however, the HIVdb scores were consistently higher for most antiretroviral drugs when DEEPGEN™ was used with a mutation frequency of ≥ 1% (Fig. 2b). In fact, no significant differences were observed in the HIVdb scores determined by Sanger or DEEPGEN™ using mutation frequencies ≥ 20% when all drug classes (PI, NRTI, NNRTI, and INSTI) were compared for both cohorts of patients (Fig. 2c). As expected, significantly higher HIVdb scores were obtained -for all antiretroviral drugs- using DEEPGEN™ with mutation frequencies ≥ 1% compared to Sanger or DEEPGEN™ with mutation frequencies ≥ 20%, i.e., 26- and twofold (PI), 16- and sixfold (NRTI), four- and twofold (NNRTI), and four- and twofold (INSTI) for St. George’s and NHS Lothian, respectively (Paired *t* test, *p* < 0.01 to *p* < 0.0001) (Fig. 2c).

**Fig. 3:**
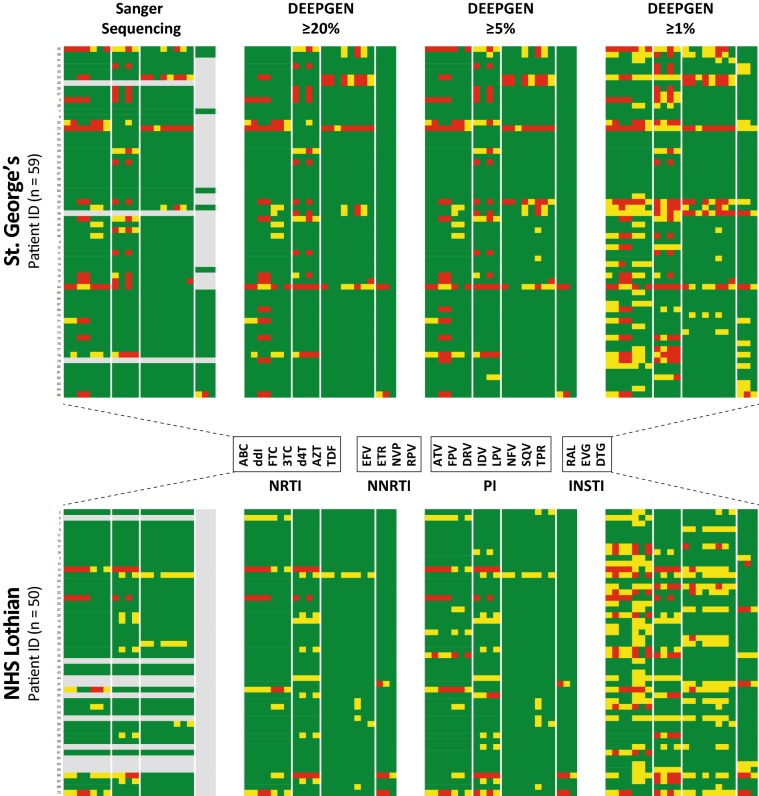
HIV-1 genotypic resistance interpretation based on Sanger sequencing or DEEPGEN™ at three thresholds (≥ 1%, ≥ 5%, and ≥ 20%) A list of all the amino acid substitutions (mutations) determined for all 109 viruses with each assay and condition were used with the HIVdb Program Genotypic Resistance Interpretation Algorithm from the Stanford University HIV Drug Resistance Database (http://hivdb.stanford.edu) to infer the levels of susceptibility to PIs, NRTIs, NNRTI,s and INSTIs. High-level and intermediate resistance profiles are indicated in red and yellow, respectively; while a susceptible genotype is depicted in green. Resistance profiles not obtained (Sanger sequencing not performed) are indicated in grey. All 109 HIV-infected individuals from both cohorts of patients are indicated. NRTI (abacavir, ABC; didanosine, ddI; emtricitabine, FTC; lamivudine, 3TC; stavudine, d4T; tenofovir, TDF; zidovudine, AZT), NNRTI (efavirenz, EFV; etravirine, ETR; nevirapine, NVP; rilpivirine, RPV), PI (atazanavir, ATV; darunavir, DRV; amprenavir, APV; indinavir, IDV; lopinavir, LPV; nelfinavir, NFV; saquinavir, SQV; tipranavir, TPV), and INSTI (dolutegravir, DTG; elvitegravir, EVG; raltegravir, RAL)

Although the clinical significance of minority variants is still on debate, here we assumed equal impact on predicted drug resistance of a mutation detected by Sanger sequencing or DEEPGEN™ with mutation frequency thresholds of ≥ 20%, ≥ 5%, or ≥ 1%, the HIVdb scores to infer the levels of susceptibility to the different antiretroviral drugs. As observed in Fig. 3, similar drug resistance profiles (susceptible, low-level/intermediate, or high-level resistance) were obtained using Sanger and DEEPGEN™ with mutation frequencies ≥ 20%; however, a few additional mutations detected at frequencies between 5 and 20% increased the resistance level to certain NRTIs, NNRTIs and/or PIs in viruses from seven St. George’s and nine NHS Lothian’s patients. This effect was more dramatic when all mutations with frequencies ≥ 1% were included. A total of 25 (42.4%) and 21 (42%) HIV-infected individuals in the St. George’s and NHS Lothian cohorts, respectively, showed some kind of resistance HIV-1 genotype (reduced susceptibility) determined by Sanger or DEEPGEN™ with mutation frequencies ≥ 20%; however, these numbers increased to 44/59 (74.6%) and 42/50 (84%) using DEEPGEN™ with mutation frequencies ≥ 1% (Fig. 3), which correlated with the antiretroviral drugs listed in their treatment histories (Supp. Tables 1 and 2).

**Fig. 4:**
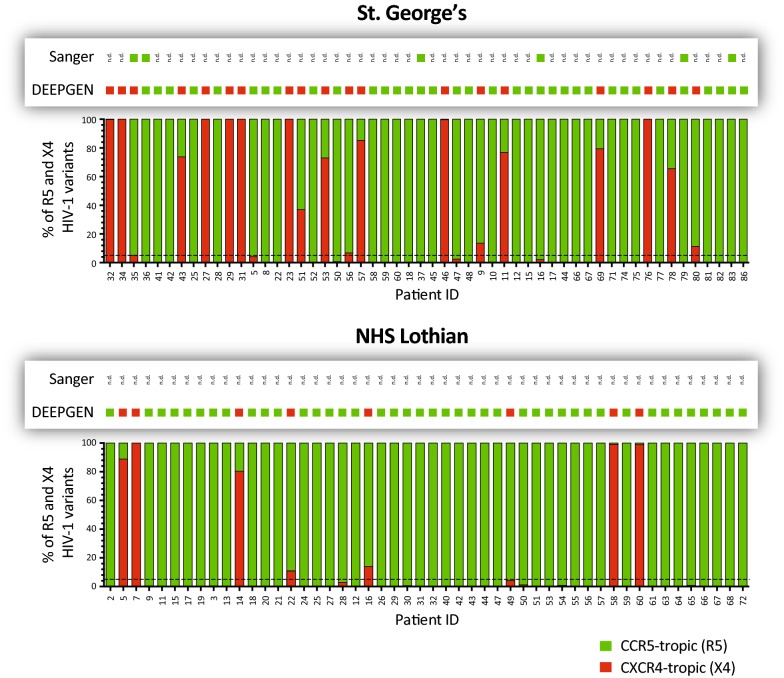
HIV-1 coreceptor tropism determination using Sanger sequencing or DEEPGEN™. The percentage of CCR5- (R5) or CXCR4- (X4) tropic HIV-1 variants in each patient -determined with DEEPGEN™- is indicated in the plots. Green and red blocks in the boxes above each plot (patient cohort) indicate the absence or presence of non-R5 (X4) viruses, respectively, as determined by Sanger sequencing or DEEPGEN™. n.d., not determined

Finally, we used DEEPGEN™ to quantify the frequency of CCR5- or CXCR4-tropic variants and determine HIV-1 coreceptor tropism in both cohorts of patients. HIV-1 tropism based on Sanger sequencing had been determined in six patients from St. George’s, all classified as being infected with R5 viruses (Fig. 4). Interestingly, DEEPGEN™ was able to corroborate the R5 tropism in 5/6 viruses while in one patient X4 HIV-1 variants were detected at low frequency (i.e., 5.1%), changing the tropism determination to dual- or mixed-tropic (D/M-tropic). Overall, 35% of the St. George’s patients were infected with D/M-tropic viruses, with the frequency of X4 variants ranging from 5.1 to 100% within the HIV-1 population (Fig. 4). In the case of the NHS Lothian, only 16% (8/50) of the patients harbored D/M-tropic HIV-1 strains, with X4 variants ranging from 10.9 to 100% (Fig. 4).

### Verifying DEEPGEN™ in the United Kingdom

**Fig. 5:**
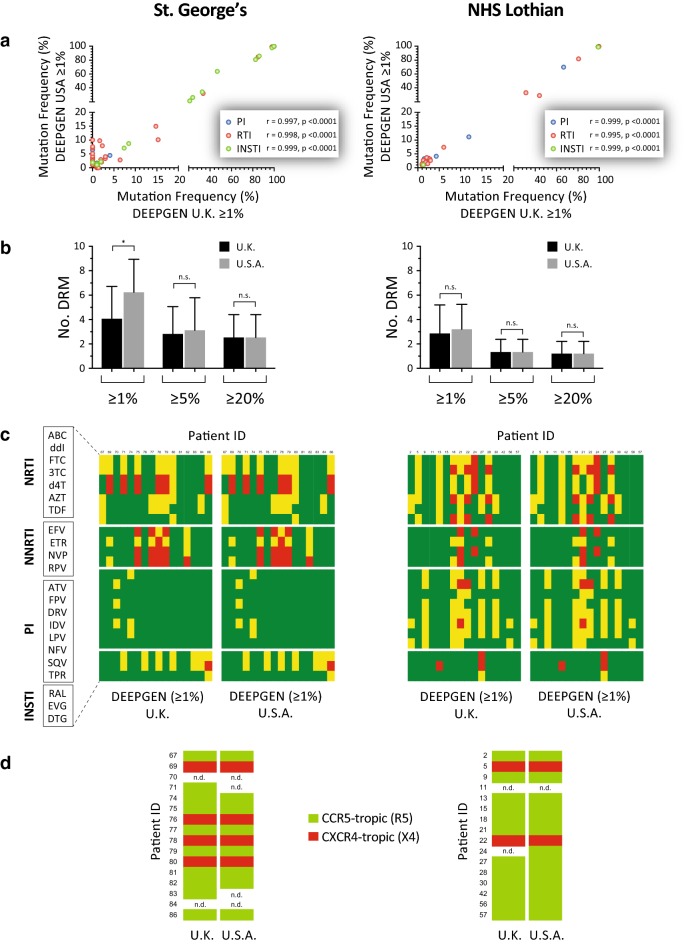
Validation of DEEPGEN™ in the U.K. Sixteen plasma samples from each cohort of patients originally analyzed with DEEPGEN™ in the respective clinical laboratory (St. George’s and NHS Lothian) was sent to Cleveland, OH to be re-tested with DEEPGEN™ in the UHTL. The frequency and number of drug resistance mutations detected in the U.S. and the respective clinical laboratory in the U.K. are indicated in panels (**a**) and (**b**), respectively. Comparisons of the HIV-1 genotypic interpretation and coreceptor tropism determination are depicted in panels (**c**) and (**d**). See “Methods” section and figures legends for Figs. 2, 3, and 4 for more details on the study designs

Following the implementation of DEEPGEN™ in the clinical laboratories at St. George’s and NHS Lothian, and the successful test of 59 and 50 clinical HIV-1 samples in the respective institutions, 32 of these samples (16 from each group) were sent to the University Hospitals Translational Laboratory (UHTL, Cleveland, Ohio, USA) to complete the verification of the assay in the U.K. laboratories. After testing all 32 samples with DEEPGEN™ in the UHTL, we quantified the number of mutations -and their frequency in the population- determined in the UHTL and compared them to the values obtained in the U.K. (St. George’s and NHS Lothian). As expected, strong significant correlations were observed when the two sets of 16 sequences were compared, even after segregating the mutations per drug class, with *r* values ranging from 0.995 to 0.999 (*p* < 0.0001, Pearson coefficient correlation) (Fig. 5a). We next quantified the number of drug resistance mutations detected using different mutation frequency thresholds for DEEPGEN™ (≥ 1%, ≥ 5%, or ≥ 20%) in all three laboratories. With the exception of a slight difference between St. George’s and UHTL in the number of drug resistance mutations quantified at ≥ 1% (mean 4.1 vs. 6.2 mutations, *p* = 0.029, Mann–Whitney), no significant differences were observed when the number of mutations associated with drug resistance were quantified in the U.S. or in the U.K. (Fig. 5b). More importantly, no difference was observed in the drug resistance profiles determined using the HIVdb algorithm (Fig. 5c). Finally, a perfect agreement (100% concordance, κ = 1) was observed comparing HIV-1 coreceptor tropism determinations based on V3 sequences obtained in the U.K. (St. George’s and NHS Lothian) and in the U.S. (UHTL) (Fig. 5d).

### Minority HIV-1 drug resistant variants in patients from the United Kingdom

**Fig. 6:**
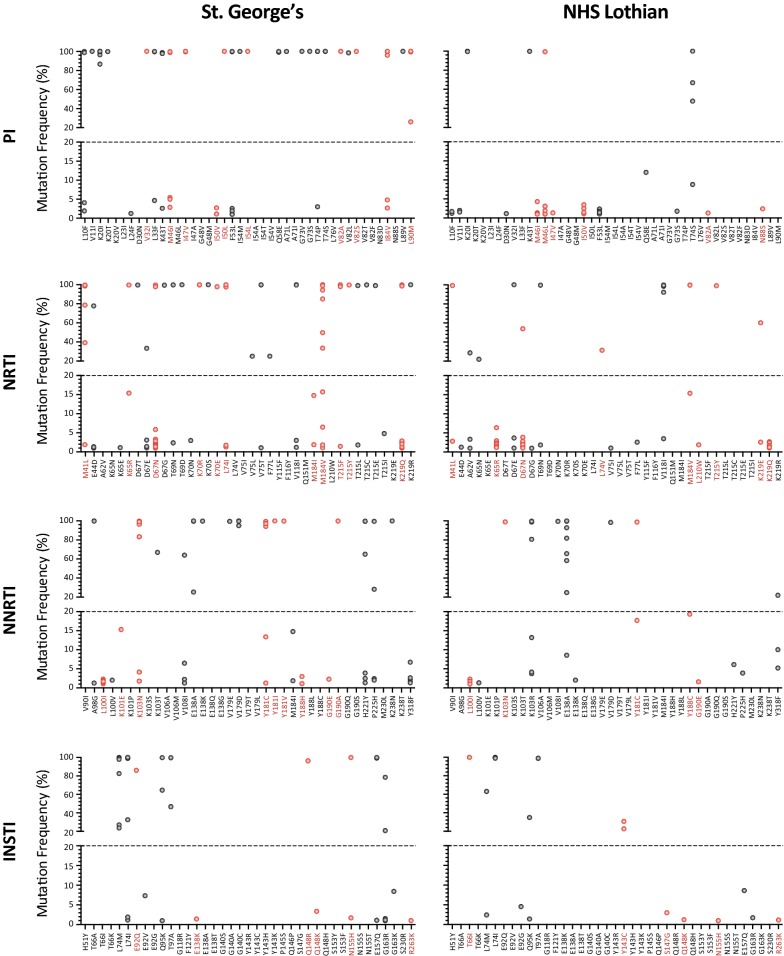
Number and frequency of HIV-1 drug resistance mutations in all 109 patients from the St. George’s and NHS Lothian cohorts quantified using DEEPGEN™. Primary and secondary/compensatory drug resistance mutations, defined by the Stanford University HIV Drug Resistance Database (http://hivdb.stanford.edu), are indicated as red and grey dots, respectively. The four panels summarize the amino acid substitutions (mutations), and their frequencies within the population, associated with resistance to PIs, NRTIs, NNRTIs, and INSTIs identified in any of the 109 patients

As described above, we implemented and verified the use of our deep sequencing-based HIV-1 genotyping and coreceptor tropism assay (DEEPGEN™) in the U.K. to study minority HIV-1 drug resistant variants in patients from London (St. George’s) and Edinburgh (NHS Lothian). Figure 6 summarizes all the primary and compensatory drug resistance mutations, and their frequency in the HIV-1 quasispecies, identified by DEEPGEN™. As expected, the number and type of mutations with frequencies > 20% (those within Sanger sequencing-based detection level) matched the cART history of the patient, e.g., the virus from patient SG28 had mutations associated with resistance to ABC (L74I 97.6% frequency), 3TC (M184V 99.7%), and EFV (K103N 98.8%, P225H 99.5%) and had been exposed to RTV, DRV, ABC, 3TC, and EFV; while patient SG86 had a virus with resistance to FTC (M184V 98.9%) and RAL (L74M 97.9%, E92Q 86.1% and T97A 99.8%) after being treated over the years with RTV, LPV, DRV, FTC, TDF, and RAL (Additional file 1: Table S1). Similar drug resistance profiles were observed for individuals experiencing virologic failure from both cohorts of patients (data not shown). More interestingly, a number of minority mutations at frequencies below the Sanger threshold (~ 20%) were detected in a multitude of individuals (Fig. 6). In the 59 patients from St. George’s, 87, 126, 66, and 55 (334 total) mutations associated with resistance to PIs, NRTIs, NNRTIs, or INSTIs were detected, respectively; of those, 18, 68, 31, and 14 (131 total) were present at frequencies below 20%. That means that approximately 40% (131/334) of the total number of drug resistance mutations from these patients were present in minority HIV-1 variants. On the other hand, in the NHS Lothian cohort with a higher number of cART-naïve patients (Table 1), we observed a reduced number of drug resistance mutations compared to the St. George’s cohort (167 vs. 334, respectively), although with a slightly higher proportion of drug resistance minority mutations, i.e., 66% (111/167) (Fig. 6), most likely a consequence of the reduced number of majority (> 20% frequency) drug resistance mutations identified in this cohort of patients.

The distribution of minority drug resistance mutations per drug class was different in both cohorts of patients. A higher proportion of minority PI-, NRTI-, and INSTI-resistance mutations was detected in NHS Lothian patients compared to individuals from St. George’s, i.e., 33/41 (80.5%) vs. 18/87 (20.7%), 53/70 (75.7%) vs. 68/126 (54%), and 9/21 (42.9%) vs. 14/55 (25.5%), respectively (unpaired *t* test, *p* < 0.0001) (Fig. 6). The most prevalent primary minority drug resistance mutations observed in both cohorts of patients were the PIs M46I/L (in 3 and 9 SG and NHS individuals, respectively) and I50 V (2 and 5); the NRTIs D67N (23 and 20), K65R (0 and 9), L74I (2 and 0), M184V/I (6 and 0) and K219Q (9 and 10); and the NNRTI L100I (6 and 3). Although a few minority primary and compensatory INSTI mutations were observed in viruses from both cohort of patients, none of them were observed at a particularly relevant prevalence (Fig. 6). Most of these minority drug resistance mutations were detected in the 67 cART-experienced individuals, e.g., the HIV-1 genotype of the following patients consisted of a mixture of majority and minority drug resistance mutations: patient SG79 (PR V11I 99.9%, K20I 99.9%; RT E44D 1.3%, D67N 1.1%, L100I 1.7%, M184V 84.9%, M184I 14.8%, K219Q 2.1%, INT E157Q 99.8%), patient SG86 (RT M184V 99.8%, INT L74M 97.9%, L74I 1.9%, E92Q 86.1%, E92V 7.4%, T97A 99.8%, G163R 1.1%), patient NHS49 (PR E35G 1.3%, M46L 3.1%, I50 V 2.5%, F53L 1.4%, RT M41L 99.2%, D67N 2.3%, M184V 15.4%, T215Y 99.1%) and patient NHS72 (PR M46L 1.1%, RT D67N 1.4%, K103R 99.6%, M184V 99.8%, Y188C 19.3%, INT T66I 99.8%, L74I 99.8%, T97A 98.8%, E157Q 8.7%). On the other hand, 27 of the 42 cART-naïve patients carried viruses with a mixture of primary and compensatory minority drug resistance mutations. A range of 1–3 minority mutations were detected in half of the cART-naïve individuals in the St. George’s cohort (7/14). Interestingly, a higher proportion of cART-naïve NHS Lothian patients (20/28) had viruses with minority drug resistance mutations, ranging from 1 to 8 mutations per patient. Most of the minority mutations in viruses from both groups of naïve patients were observed in the RT, e.g., M41L, E44D, A62V, K65R, D67N, D67G, V75I, L100I, K103N, K103R, V188I, M184I, L210W, K219Q, Y318F, etc., although a number of minority mutations associated with resistance to PI (L10F, V11I, M46I/L, I50V, F53L, Q58E, T74S) or INSTI (L74I/M, E92G, E138K, S147G, G163R, Q148K) were also identified (Fig. 6). Finally, it is interesting to highlight that most HIV-1 drug resistance mutations were observed at the ends of the spectrum in the HIV-1 population, with only a small fraction of mutations detected at frequencies between > 5 and < 90% (Fig. 6). This was more accentuated in PI and NRTI mutations from both group of viruses, ranging from 1/87 to 4/41 mutations in this mutation frequency range, while a slightly higher number of NNRTI and INSTI mutations were observed in this “transitional phase”, ranging from 8/66 to 12/35, respectively (Fig. 6).

### Phylogenetic and diversity analysis using deep sequencing

**Fig. 7:**
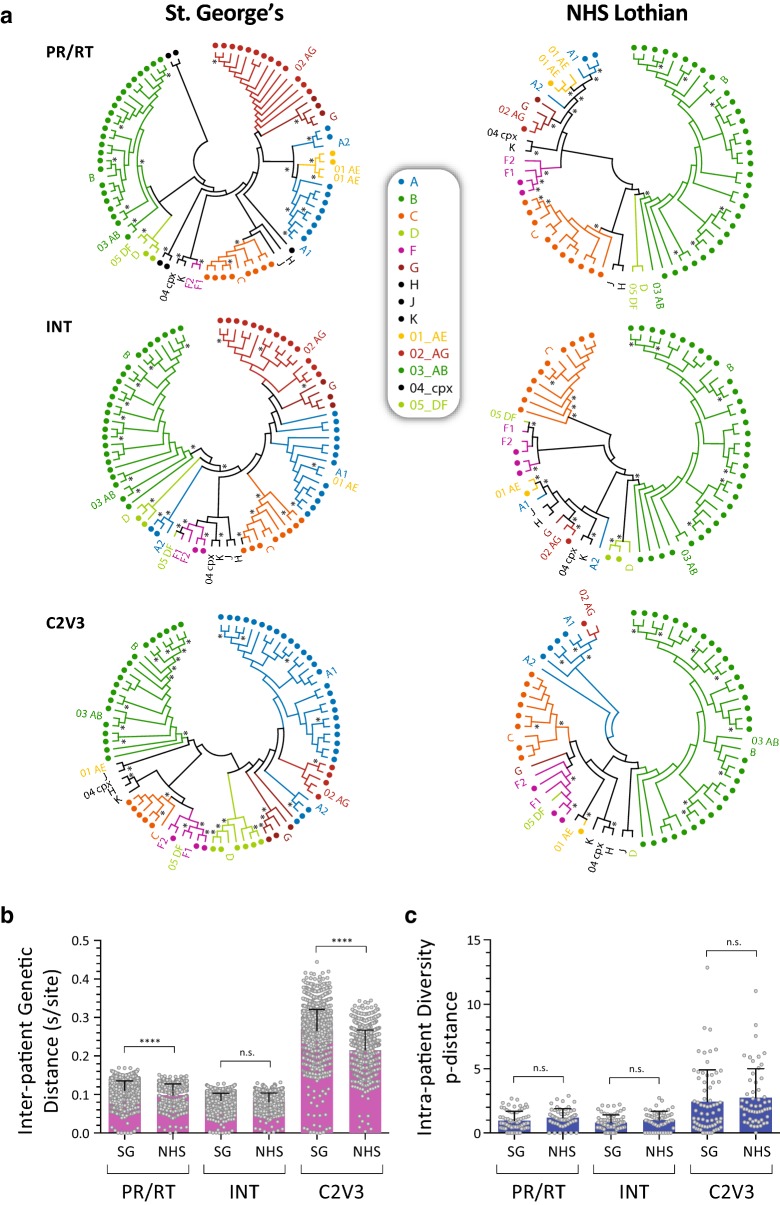
Phylogenetic and HIV-1 genetic diversity analysis. **a** Neighbor-joining phylogenetic trees constructed using consensus sequences generated with DEEPGEN™ corresponding to the HIV-1 protease and reverse transcriptase (PR/RT), integrase (INT), and C2V3 coding regions from the 59 and 50 patient-derived viruses from St. George’s and NHS Lothian cohorts, respectively. Sequences from 16 viruses from different Group M HIV-1 subtypes, obtained from Los Alamos HIV Sequence Database (https://www.hiv.lanl.gov/content/sequence/HIV/mainpage.html), were used as reference, i.e., A1.AU.03.PS1044_Day0.DQ676872, A2.CD.97.97CDKTB48.AF286238, B.FR.83.HXB2_LAI_IIIB_BRU.K03455, C.BR.92.BR025_d.U52953, D.CD.83.ELI.K03454, F1.BE.93.VI850.AF077336, F2.CM.02.02CM_0016BBY-AY3711158, G.BE.96.DRCBL.AF084936, H.BE.93.VI991.AF190127, J.CD.97.J_97DC_KTB147.EF614151, K.CD.97.97ZR_EQTB11.AJ249235, CRF01_AE.AF.07.569M.GQ477441, CRF02_AG.CM.99.pBD6_15.AY271690, CRF03_AB.RU.97.KAL153_2.AF193276, CRF04_cpx.CY.94.94CY032_3.AF049337, and CRF05_DF.BE.93.VI961.AF076998. Bootstrap resampling (1000 data sets) of the multiple alignments tested the statistical robustness of the trees, with percentage values above 75% indicated by an asterisk. Each dot represents a patient-derived consensus sequence. **b** HIV-1 inter-patient genetic distances determined using the Maximum Composite Likelihood model with bootstrap as the variance estimation method (1000 replicates) within MEGA 6.06 [45]. **c** Intra-patient HIV-1 quasispecies diversity determined using all three PR/RT-, INT-, and C2V3- coding regions based on the p-distance model as described for deep sequencing [46]. Means ± standard deviations and statistically significant differences between both cohorts of patients (unpaired t test) are marked by ****(*p *< 0.0001), ***(*p* < 0.001), **(*p* < 0.01), *(*p* < 0.05), and n.s. (*p* > 0.05). n.d., not determined; SG, St. George’s; NHS, NHS Lothian

As described above, DEEPGEN™ is based on deep sequencing viral RNA extracted from plasma samples and optimized to accurately detect minority HIV-1 variants above a 1% frequency level in the HIV-1 population [19]. Moreover, this methodology is capable of generating over 10,000 HIV-1 sequences (reads) per patient that can be used to analyze inter- and intra-patient HIV-1 genetic diversity [11, 41, 49]. Phylogenetic analyses confirmed the HIV-1 subtype initially determined for each patient-derived virus with the DEEPGEN™ Software Tool Suite v2 and Geno2Pheno. HIV-1 subtyping classification varied slightly depending on the HIV-1 genomic region analyzed (*pol* or C2V3); however, based on the most broadly used C2V3 region [19, 50, 51], the HIV-1 subtypes identified in these patients included: A1 (20), B (15), C (7), CFR02_AG (6), G (4), A2 (3), D (2), and F1 (2) in St. George’s cohort and B (33), C (8), A1 (4), F1 (3), CRF02_AG (1), and CFR01_AE (1) in NHS Lothian’s patients (Fig. 7a and Additional file 1: Table S1, Additional file 2: Table S2).

As expected, mean inter-patient genetic distances calculated with the consensus C2V3 sequences were higher than those calculated with the PR/RT and INT sequences (Fig. 7b). Viruses from St. George’s patients were significantly more diverse than viruses from NHS Lothian’s individuals comparing C2V3 (mean 0.265 vs. 0.215 substitutions per site, *p* < 0.0001 unpaired t test) and PR/RT (0.111 vs. 0.098 s/site, *p* < 0.0001 unpaired t test) sequences but not when comparing INT sequences (0.084 vs. 0.081 s/site, *p* = 0.082 unpaired t test), respectively (Fig. 7b). Finally, intra-patient HIV-1 diversity was also determined using all three PR/RT-, INT-, and C2V3- coding regions based on the p-distance model as described for deep sequencing [46]. Although slightly higher in patients from NHS Lothian compared with viruses from St. George’s individuals, no significant difference was observed in the HIV-1 quasispecies diversity of these patients, i.e., PR/RT (1.212 vs. 0.968), INT (1.013 vs. 0.817), and C2V3 (2.761 vs. 2.446), respectively (Fig. 7c).

### Association of DEEPGEN™-based HIV-1 genotyping with clinical parameters

Given the considerable amount of data that we were able to accumulate from deep sequencing patient-derived HIV-1 sequences from these HIV-infected individuals, i.e., majority (frequency > 20%) and minority (frequency < 20% and > 1%) drug resistance mutations, susceptibility to antiretroviral drugs (HIVdb scores), coreceptor tropism, subtyping, inter-patient and intra-patient viral diversity, we decided to investigate potential associations among any of these metrics and clinical parameters, mainly plasma HIV RNA load, CD4^+^ T-cell counts, and antiretroviral therapy history. As expected, HIVdb scores determined using only majority, or including minority, drug resistance mutations correlated significantly with ART history in patients from St. George’s (*r* = 0.51, *p* < 0.0001 or *r* = 0.58, *p* < 0.0001 Pearson coefficient correlation) and NHS Lothian (*r* = 0.37, *p* = 0.007 or *r* = 0.45, *p* = 0.001). On the other hand, no significant association was observed in most pairwise comparisons of the multiple virological metrics and clinical parameters studied (data not shown). For example, no significant correlation was observed between HIVdb scores determined using only majority (*r* = 0.13, *p* = 0.30 or *r* = 0.01, *p* = 0.98) or including minority (*r* = 0.12, *p* = 0.38 or *r* = 0.10, *p* = 0.47) drug resistance mutations, ART history (*r* = 0.22, *p* = 0.08 or *r* = 0.13, *p* = 0.35) or intra-patient HIV-1 diversity (*r* = 0.11, *p* = 0.39 or *r* = 0.10, *p* = 0.46) with plasma HIV RNA load in individuals from St. George’s or Lothian cohorts. However, inverse significant correlations were observed between the number of drugs in the ART history (*r* = − 0.22, *p* = 0.01 and *r* = − 0.43, *p* = 0.001) or intra-patient HIV-1 diversity (*r* = − 0.30, *p* = 0.01 and *r* = − 0.26, *p* = 0.01) and CD4^+^ T-cell counts in St. George’s and Lothian patients, respectively (data not shown).

## Discussion

Widespread HIV-1 drug resistance, usually associated with suboptimal virological suppression and poor clinical outcomes [52, 53], is the natural byproduct of years of treating HIV-infected individuals with cART. Monitoring and detecting HIV-1 drug resistance, as soon as possible, does not only help control the infection and preserve the immunologic response in the individual but also limits the transmission of HIV-1 drug resistant variants, restricting the increasing prevalence of pretreatment resistance [53, 54]. Deep sequencing-based HIV-1 genotyping assays have the intrinsic capability of detecting minority HIV-1 drug resistant variants before they become majority members of the HIV-1 quasispecies, which may lead to virologic failure [11, 19, 39, 55, 56]. Thus, the use of these highly sensitive assays should help controlling HIV-1 drug resistance both at the individual (patient) and population (epidemic) levels. In this study, we evaluated the use of DEEPGEN™, a deep sequencing-based HIV-1 genotyping and coreceptor tropism assay implemented in the clinical setting in the United States since 2013 [19] and in Uganda since 2017 [41], in two clinical laboratories in the U.K. i.e., St. George’s University Hospitals Healthcare NHS Foundation Trust (London) and at NHS Lothian (Edinburgh). As expected, DEEPGEN™ was able to accurately detect a series of drug resistance-associated mutations not identified using standard Sanger sequencing-based tests, correlating significantly with the patient’s cART history and providing a more accurate characterization of drug resistant HIV-1 infections in these clinical institutions.

Adapting and implementing deep sequencing-based methodologies has become much easier and accessible since its inception in the early 2000s [11]. A multitude of deep sequencing-based tests have been developed and are being offered in clinical laboratories aimed to asses genomic, cancer, or infectious diseases related conditions [11, 57–59] and HIV/AIDS is not the exception. Still, while numerous groups have used these methodologies in research studies, only a few deep sequencing-based HIV-1 tests have been developed to be used in nationally accredited (to ISO 15189 standards) and CLIA/CAP-accredited laboratories [19, 27, 28]. Several studies have compared the performance of deep versus Sanger sequencing for HIV-1 genotypic resistance testing [10, 19, 25, 28, 39]; however, this is the first study evaluating the implementation of DEEPGEN™, a clinically validated deep sequencing-based HIV-1 genotyping assay, in two clinical laboratories in the U.K. A previous study had described a limited evaluation of the now obsolete Roche 454 HIV-1 ultradeep sequencing drug resistance assay at Royal Free London NHS Foundation Trust [60]. Here, both clinical laboratories (St. George’s and NHS Lothian) were already equipped with the proper instrumentation to perform deep sequencing (i.e., Ion Torrent’s PGMs) and were able to successfully perform DEEPGEN™ in their own facilities by following the Standard Operating Procedures developed at the UHTL (Cleveland, OH). The quality of all PGM runs in the U.K. were comparable, if not better, to those performed in the U.S., generating excellent deep sequencing run metrics (e.g., coverage, quality reads, median read lengths, etc.) confirming the established quality assurance of the system. This led to a perfect correlation during the verification studies, where a series of plasma samples from HIV-infected individuals were evaluated in parallel in the U.K. (St. George’s and NHS Lothian) and U.S. (UHTL) laboratories, i.e., the number of drug resistance associated mutations, drug resistance profiles (HIVdb scores) and HIV-1 coreceptor tropism determinations matched 100%, underscoring the capability of both U.K. clinical laboratories to perform the assay on site.

Similar to previous studies [19, 41, 49, 61], DEEPGEN™ detected all the drug resistance mutations, in all 109 patients, originally identified in each laboratory using Sanger sequencing. More importantly, a total of 280 additional drug resistance mutations were identified in both cohorts of HIV-infected individuals, i.e., mutations below the limit of detection of Sanger sequencing (~ 20%) [14–18] and only detectable using deep sequencing, therefore modifying the Sanger-based HIVdb scores and overall resistance interpretation. The kind, number, and frequency of the minority drug resistance mutations identified matched the cART history of the patients, the most common being M46I/L and I50 V (PIs), K65R, D67 N, L74I, M184 V/I, and K219Q (NRTIs), and L100I (NNRTIs). A few minority INSTI-resistance mutations were observed in the 109 HIV-infected individuals, reflecting the limited number of patients being treated with INSTIs at the time of the study (23/109). Most of these mutations have also been detected as minority variants in cohorts of patients failing first- or second-line cART [10, 40, 41, 49, 55, 61–64] or in antiretroviral-naïve patients [10, 65–69], including a study from the U.K. [3]. As expected, drug resistance profiles based on Sanger sequencing correlated significantly with cART history; however, the correlation was stronger when minority mutations were included in the analysis, suggesting that the presence of drug resistant minority variants as part of the HIV-1 quasispecies is a direct consequence of the antiretroviral drug pressure. Interestingly, minority drug resistant variants were observed in both antiretroviral-experienced and antiretroviral-naïve individuals, some of them associated with the current cART of each patient but others not related nor conferring cross-resistance to any particular drug in the respective regimens. These minority variants may be lurking in the population, waiting for the proper conditions to be selected [20, 22]. However, based on our cross-sectional analysis, it is difficult to discern whether the increase in drug resistance (mutations, HIVdb scores, resistance profiles) due to the detection of minority variants at the time the plasma samples were obtained will result in an increase in plasma viremia and subsequent immunologic decline.

It is important to highlight that DEEPGEN™, in addition to determining HIV-1 drug resistance and coreceptor tropism, was also designed to evaluate subtyping, inter-patient and intra-patient HIV-1 diversity based on *pol* and *env* genes [19]. Here we were able to assess all these viral parameters for all 109 HIV-infected individuals. Interestingly, while 44% (48/109) of the patients in this study were infected with subtype B HIV-1 strains (66% in NHS Lothian’s patients), several non-B HIV-1 strains were detected in these individuals, including A1, A2, C, D, F1, G, CRF02_AG, and CRF01_AE. Most of these non-B HIV-1 subtypes have been previously reported in the U.K. [4, 70–72]; however, it is important to highlight the presence of three individuals infected with subtype F1 HIV-1 strains. Prevalence of subtype F1 viruses has been increasing in North East Spain, particularly among men who have sex with men [73, 74]. Since response to cART seems to be impaired in patients infected with F1 viruses [74, 75], it will be important to monitor the circulation of this HIV-1 subtype in the U.K., particularly with the recent increase in chemsex among MSM living with HIV-1 in the country [76].

As described above, a number of studies -including some from the U.K.—have shown that deep sequencing assays are excellent tools to increase the detection of drug resistance mutations [19, 34, 40, 41, 62, 63, 65, 67], monitor transmission of HIV-1 drug resistance [3, 19, 33, 41, 49, 61, 63, 65, 68, 69, 77–82], and potentially determine the relevance of detecting minority drug resistance mutations in the clinical setting [10, 11, 31, 40, 55, 83, 84]. Are these minority drug resistant HIV-1 variants going to be selected as majority members of the quasispecies, eventually contributing to elevated plasma viremia and leading to virologic failure? What is the real importance and/or clinical significance of these minority variants? A multitude of studies have attempted to address these questions, adding to the controversy [29–33, 40, 41]. Although it may seem logical that, under the right (drug) pressure, these drug resistant minority variants will become majority members of the HIV-1 population, only a few studies have been able to clearly demonstrate that pre-existent minority variants contribute to a negative clinical outcome [33, 41, 62, 69, 85]. It is clear that further studies based on larger and well-characterized cohorts of patients, and using clinically validated deep sequencing-based HIV-1 genotyping assays such as DEEPGEN™, will be needed to determine whether drug resistant minority variants contribute to virologic failure.

In summary, to our knowledge, this is the first study evaluating the transition, training, and implementation of DEEPGEN™, a deep sequencing-based HIV-1 genotyping assay, between three clinical laboratories in two different countries (we are in the process of publishing the establishment of DEEPGEN™ in Uganda). More importantly, we were able to characterize the HIV-1 drug resistance profile (including minority variants), coreceptor tropism, subtyping, and intra-patient viral diversity in 109 individuals from the United Kingdom, providing valuable information to help control the HIV/AIDS epidemic in the country. This study provides a rigorous basis for basing clinical decisions on highly sensitive and cost-effective deep sequencing-based HIV-1 genotyping assays. Moreover, our work is an example of a verification study of a fully validated deep sequencing-based HIV-1 genotyping assay, which can replace Sanger sequencing assays and improve the HIV-1 drug resistant profiles of HIV-infected patients. DEEPGEN™ can be effectively implemented into nationally accredited clinical and molecular pathology laboratories in the U.K., supporting local HIV-1 treatment services and contributing to public health programs that monitor the emergence and transmission of HIV-1 drug resistance quasispecies in the country.

## Additional files

**Additional file 1: Table S1.** Demographic, clinical and virological characteristics from St. George’s patients.

**Additional file 2.** Demographic, clinical and virological characteristics from NHS Lothian’s patients.
