# Supplementary material for: Characterization of minority HIV-1 drug resistant variants in the United Kingdom following the verification of a deep sequencing-based HIV-1 genotyping and tropism assay
Source: AIDS Res Ther. 2018 Nov 8;15:18. doi: 10.1186/s12981-018-0206-y (PMC6223033; doi:10.1186/s12981-018-0206-y)
Supplement: Supplementary file 1 — Additional file 1: Table S1. Demographic, clinical and virological characteristics from St. George’s patients. [file 12981_2018_206_MOESM1_ESM.docx]

**Additional File 1: Table S1**. Demographic, clinical and virological characteristics from St. George’s patients

| Patient ID | Sex ^a^ | Age ^b^ | Risk Factor ^c^ | Sample Date ^d^ | Plasma HIV-1 RNA (log_10_ c/ml) ^e^ | CD4^+^ T cells (cell/mm^3^) ^f^ | Subytpe *pol*/V3 ^g^ | ART-naïve or experienced ^h^ | Treatment History ^i^ |
| --- | --- | --- | --- | --- | --- | --- | --- | --- | --- |
| 5 | F | 47 | n.d. | 11/27/14 | 5.16 | 490 | G/B | Experienced | FTC, TDF |
| 7 | M | 60 | n.d. | 12/17/14 | 6.82 | 346 | C/B | Naïve | NONE |
| 8 | M | 72 | n.d. | 12/16/14 | 4.76 | 488 | B/B | Experienced | FTC, TDF, EFV, |
| 9 | F | 41 | n.d. | 11/21/14 | 4.71 | 87 | A2/A2 | Experienced | FTC, TDF, RTV, LPV, DRV |
| 10 | F | 40 | n.d. | 12/01/14 | 5.28 | 87 | A2/A2 | Experienced | DTG, ABC, 3TC, |
| 11 | M | 35 | n.d. | 01/02/15 | 4.82 | 663 | A1/A1 | Naïve | NONE |
| 12 | F | 30 | n.d. | 01/08/15 | 4.03 | 157 | C/AG | Experienced | RTV, LPV, TDF, EVG, FTC |
| 14 | M | 30 | n.d. | 01/19/15 | 4.73 | 545 | B/B | Naïve | NONE |
| 15 | M | 41 | n.d. | 01/19/15 | 4.44 | 162 | B/B | Experienced | TDF, FTC, DRV, RTV, LPV, |
| 16 | F | 50 | n.d. | 09/02/14 | 3.80 | 299 | B/B | Experienced | d4T, 3TC, NVP, ABC, RTV, LPV, DRV |
| 17 | M | 53 | HET | 08/14/14 | 4.51 | 14 | B/B | Experienced | ABC, DRV, RAL |
| 18 | M | 44 | n.d. | 07/09/14 | 6.57 | 453 | B/F1 | Experienced | RTV, DRV, RAL, TDF, FTC |
| 20 | M | 59 | n.d. | 03/22/12 | 6.14 | 85 | B/A1 | Experienced | EFV, FTC, TDF, RTV, LPV, DRV |
| 22 | F | 19 | n.d. | 10/06/09 | 3.88 | 342 | AG/B | Experienced | FTC, TDF, ATV, 3TC, RTV, LPV, MVC |
| 23 | F | 42 | n.d. | 12/10/09 | 3.52 | 679 | A1/A1 | Experienced | AZT, d4T, ddI, 3TC, NVP, NFV, RTV, IDV, DLV, ABC, ATV, LPV, FTC, TDF, RAL, ETR |
| 24 | F | 20 | n.d. | 07/14/10 | 5.72 | 78 | AG/A1 | Experienced | 3TC, ddI, d4T, NFV, RTV, TDF, NVP, TRV, LPV, FTC, ATV, ABC, AZT |
| 25 | F | 20 | n.d. | 03/03/09 | 5.11 | 189 | AG/A1 | Experienced | 3TC, ddI, d4T, NFV, RTV, TDF, NVP, TRV, LPV, FTC, ATV, ABC, AZT |
| 26 | M | 45 | n.d. | 10/01/10 | 3.18 | 310 | AG/A1 | Experienced | 3TC, AZT, EFV, NVP, TDF, RTV, LPV, SQV, ATV, FTC, DRV |
| 27 | F | 30 | n.d. | 10/30/09 | 3.59 | 539 | B/B | Experienced | RTV, LPV, 3TC, AZT, EFV, ABC, ETR |
| 28 | M | 59 | n.d. | 09/22/09 | 4.46 | 171 | C/C | Experienced | ABC, EFV, 3TC, DRV, RTV |
| 39 | F | 47 | n.d. | 12/02/14 | 2.93 | 509 | B/B | Experienced | AZT, ddI, 3TC, d4T, NVP, SQV, TDF, ATV, RTV, LPV, FTC, DRV, DTG |
| 41 | F | 37 | HET | 09/16/14 | 4.91 | 205 | D/D | Experienced | RTV, LPV, ATV, FTC, TDF, DRV |
| 42 | F | 35 | n.d. | 01/07/15 | 6.42 | 158 | B/A1 | Experienced | DRV, RTV, FTC, TDF |
| 43 | M | 49 | MSM | 11/17/14 | 4.38 | 91 | C/C | Experienced | FTC, TDF, RAL, ETR, DRV |
| 44 | F | 24 | n.d. | 01/11/10 | 3.09 | 534 | C/C | Experienced | ddI, d4T, NVP, AZT, ATV, FTC, TDF, RTV, LPV |
| 45 | M | 56 | n.d. | 08/05/10 | 3.19 | 1,017 | B/A1 | Experienced | EFV,3TC, d4T, NVP, AZT, DRV, RTV, LPV, |
| 46 | F | 44 | n.d. | 02/16/10 | 3.15 | 118 | D/D | Experienced | d4T, NVP, ddI, RTV, LPV, SQV, AZT, ABC, 3TC, FTC, TDF, ATV |
| 47 | F | 45 | n.d. | 09/09/14 | 3.46 | 276 | A1/AG | Experienced | EFV, 3TC, AZT, NVP, ABC, ATV, RTV, LPV |
| 48 | M | 34 | n.d. | 10/14/14 | 6.03 | 542 | B/B | Naïve | NONE |
| 49 | F | 24 | n.d. | 09/08/10 | 3.09 | 381 | AG/G | Experienced | 3TC, AZT, NVP, ABC, DRV, RTV, LPV, FTC, TDF, DTG |
| 50 | F | 46 | n.d. | 07/29/14 | 3.94 | 509 | AG/A1 | Experienced | EVG, TDF, FTC |
| 51 | F | 40 | n.d. | 10/27/14 | 5.20 | 35 | AG/A1 | Experienced | AZT, NFV, 3TC, NVP, TDF, FTC, RTV, ATV |
| 52 | M | 39 | n.d. | 11/17/14 | 5.68 | 480 | AG/A1 | Naïve | NONE |
| 53 | M | 44 | n.d. | 07/07/14 | 6.57 | 453 | BF/F1 | Naïve | NONE |
| 54 | M | 31 | n.d. | 02/04/14 | 5.37 | 358 | B/A1 | Experienced | TDF, FTC, EFV, DRV, RTV, LPV |
| 56 | M | 38 | n.d. | 11/18/14 | 4.88 | 436 | C/C | Experienced | EFV, FTC, TDF |
| 57 | M | 49 | MSM | 11/17/14 | 4.38 | 91 | B/B | Experienced | FTC, TDF, RAL, ETR, DRV |
| 58 | F | 67 | HET | 10/31/14 | 4.23 | 297 | G/G | Experienced | FTC, TDF, RAL |
| 59 | M | 41 | MSM | 01/14/15 | 4.55 | 455 | G/G | Naïve | NONE |
| 60 | M | 25 | n.d. | 01/08/15 | 7.00 | 318 | AG/AG | Naïve | NONE |
| 65 | M | 47 | n.d. | 05/08/15 | 2.49 | 1,120 | AG/G | Experienced | ABC, 3TC, DTG |
| 66 | M | 67 | n.d. | 05/07/15 | 2.91 | 340 | C/C | Experienced | FTC, TDF, RAL |
| 67 | M | 40 | n.d. | 02/05/15 | 4.85 | 412 | AG/AG | Experienced | RTV, ATV, EFV, ABC, 3TC |
| 69 | M | 17 | n.d. | 01/08/15 | 4.25 | 373 | C/C | Experienced | ABC, 3TC, RTV, DRV, EFV |
| 70 | F | 54 | n.d. | 02/06/15 | 4.95 | 476 | AB/B | Experienced | ABC, 3TC, DTG |
| 71 | F | 44 | HET | 03/02/15 | 3.65 | 49 | AG/A1 | Experienced | FTC, TDF, RTV, DRV |
| 72 | M | 53 | n.d. | 03/09/15 | 4.78 | 510 | B/B | Naïve | NONE |
| 74 | M | 26 | n.d. | 07/21/15 | 3.16 | 820 | A1/A1 | Naïve | NONE |
| 75 | F | 37 | n.d. | 02/10/15 | 3.47 | 115 | AG/AG | Experienced | RTV, DRV, TDF, FTC, MVC |
| 76 | F | 56 | n.d. | 04/30/15 | 3.87 | 515 | A1/A1 | Naïve | NONE |
| 77 | F | 50 | HET | 02/27/15 | 3.95 | 554 | AG/A1 | Naïve | NONE |
| 78 | M | 51 | n.d. | 04/23/15 | 4.63 | 170 | A1/A1 | Experienced | d4T, AZT, NVP, RTV, SQV, 3TC, NFV, RTV, LPV, ddI, EFV, TDF, ETR, FTC |
| 79 | F | 37 | n.d. | 04/21/15 | 4.00 | 174 | AG/AG | Experienced | RTV, DRV, TDF, FTC, MVC |
| 80 | F | 56 | n.d. | 04/18/15 | 4.91 | 16 | A1/A1 | Experienced | FTC, TDF, RTV, LPV, DRV, RAL |
| 81 | M | 35 | n.d. | 02/12/15 | 4.76 | 468 | B/B | Naïve | NONE |
| 82 | F | 51 | MSM | 03/23/15 | 4.68 | 183 | C/C | Naïve | NONE |
| 83 | F | 52 | n.d. | 05/07/15 | 4.72 | 283 | AE/A2 | Experienced | 3TC, EFV, ATV, ABC, RTV, LPV, MVC, DRV |
| 84 | F | 49 | n.d. | 06/10/15 | 7.00 | 16 | A1/A1 | Experienced | FTC, TDF, DTG, ABC |
| 86 | F | 56 | n.d. | 07/15/15 | 5.34 | 94 | A1/A1 | Experienced | FTC, TDF, RTV, LPV, DRV, RAL |

^a^ M, male; F, female. ^b^ Age at the time of sampling. ^c^ Most likely mode of HIV-1 transmission: HET, heterosexual; MSM, men who have sex with men; IVDU, intravenous drug user; MTCT, mother-to-child transmission; n.d., not determined. ^d^ date the blood sample was collected. ^e^ HIV-1 RNA plasma load (log_10_ copies/ml) at the time the blood sample was obtained. ^f^ CD4^+^ T-cell count (cells/mm^3^) at the time the blood sample was obtained. ^g^ HIV-1 subtype determined using the PR/RT- (*pol*) or the V3-coding region sequences with DEEPGEN™HIV proprietary pipeline and Geno2Pheno tools (<http://www.geno2pheno.org)>. ^h^ Patients treated (experienced) or not (naïve) with combination antiretroviral therapy (cART) at the time the blood sample was obtained. ^i^ Antiretroviral drugs used in each patient: AZT, zidovudine; ddI, didanosine; d4T, stavudine; 3TC, lamivudine; ABC, abacavir; TDF, tenofovir; FTC, emtricitabine; NVP, nevirapine; DLV, delavirdine; EFV, efavirenz; ETR, etravirine; RPV, rilpivirine; SQV, saquinavir; RTV, ritonavir; IDV, indinavir; NFV, nelfinavir; APV, amprenavir; LPV, lopinavir; ATV, atazanavir; TPV, tipranavir; DRV, darunavir; RAL, raltegravir; EVG, elvitegravir; DTG, dolutegravir; MVC, maraviroc; and COBI, cobicistat.
