# Supplementary material for: Characterization of minority HIV-1 drug resistant variants in the United Kingdom following the verification of a deep sequencing-based HIV-1 genotyping and tropism assay
Source: AIDS Res Ther. 2018 Nov 8;15:18. doi: 10.1186/s12981-018-0206-y (PMC6223033; doi:10.1186/s12981-018-0206-y)
Supplement: Supplementary file 2 — Additional file 2. Demographic, clinical and virological characteristics from NHS Lothian’s patients. [file 12981_2018_206_MOESM2_ESM.docx]

**Additional File 2: Table S2**. Demographic, clinical and virological characteristics from NHS Lothian’s patients

| Patient ID | Sex ^a^ | Age ^b^ | Risk Factor ^c^ | Sample Date ^d^ | Plasma HIV-1 RNA (log_10_ c/ml) ^e^ | CD4^+^ T cells (cell/mm^3^) ^f^ | Subytpe *pol*/V3 ^g^ | ART-naïve or experienced ^h^ | Treatment History ^i^ |
| --- | --- | --- | --- | --- | --- | --- | --- | --- | --- |
| 02 | M | 54 | MSM | 5/21/15 | 4.82 | 350 | B/B | Naïve | NONE |
| 03 | M | 43 | MSM | 5/21/15 | 4.44 | 414 | B/B | Naïve | NONE |
| 05 | F | 57 | IVDU | 6/9/15 | 5.98 | 111 | B/B | Experienced | 3TC,ETR,LPV,RTV,FTC,TDF,AZT |
| 07 | M | 28 | n.d. | 10/20/14 | 5.44 | 155 | B/B | Naïve | NONE |
| 09 | M | 43 | n.d. | 11/6/14 | 4.61 | 315 | B/B | Naïve | NONE |
| 11 | M | 25 | MSM | 11/19/14 | 5.02 | 503 | B/B | Naïve | NONE |
| 12 | M | 29 | MSM | 1/22/15 | 5.10 | 656 | B/A1 | Naïve | NONE |
| 13 | M | 25 | MSM | 2/26/15 | 4.58 | 612 | B/F1 | Naïve | NONE |
| 14 | F | 23 | MTCT | 3/18/15 | 4.54 | 13 | F1/F1 | Experienced | AZT,TDF,RAL,DRV,RTV,FTC,RPV,ATV,LPV,ABC,3TC,d4T,NFV |
| 15 | F | 60 | n.d. | 12/17/14 | 6.03 | 56 | C/C | Naïve | NONE |
| 16 | M | 59 | n.d. | 12/19/14 | 4.25 | 194 | B/B | Naïve | NONE |
| 17 | M | 58 | n.d. | 1/20/15 | 3.73 | 310 | C/B | Experienced | ABC,3TC,RTV,ATV,AZT |
| 18 | F | 57 | n.d. | 1/21/15 | 5.08 | 147 | C/C | Experienced | FTC,EFV,TDF |
| 19 | F | 40 | n.d. | 1/22/15 | 5.61 | 26 | C/C | Naïve | NONE |
| 20 | M | 46 | HET | 1/28/15 | 6.62 | 428 | AE/AE | Naïve | NONE |
| 21 | M | 24 | n.d. | 2/17/15 | 3.78 | 493 | B/B | Naïve | NONE |
| 22 | F | 21 | MTCT | 2/27/15 | 4.35 | 155 | C/C | Experienced | FTC,LPV,RTV,TDF,ABC,3TC |
| 24 | M | 36 | n.d. | 3/16/15 | 3.71 | 382 | B/B | Experienced | ABC,3TC,EFV |
| 25 | M | 38 | MSM | 4/9/15 | 4.47 | 471 | B/B | Naïve | NONE |
| 26 | M | 26 | MSM | 4/13/15 | 5.35 | 246 | B/B | Naïve | NONE |
| 27 | M | 41 | n.d. | 4/22/15 | 5.04 | 161 | B/B | Naïve | NONE |
| 28 | F | 30 | n.d. | 5/8/15 | 4.24 | 112 | C/C | Naïve | NONE |
| 29 | M | 44 | MSM | 5/21/15 | 5.73 | 23 | B/B | Naïve | NONE |
| 30 | M | 23 | MSM | 5/14/15 | 4.47 | 859 | B/B | Naïve | NONE |
| 31 | M | 44 | MSM | 6/11/15 | 5.76 | 68 | C/C | Naïve | NONE |
| 32 | M | 24 | MSM | 6/12/15 | 4.08 | 443 | B/B | Naïve | NONE |
| 40 | M | 46 | n.d. | 1/25/16 | 4.52 | 204 | B/B | Experienced | FTC,RPV,TDF,ABC,3TC,EFV,AZT |
| 42 | M | 26 | n.d. | 1/27/16 | 3.93 | 545 | B/B | Naïve | NONE |
| 43 | M | 35 | n.d. | 1/29/16 | 5.19 | 299 | A1/A1 | Naïve | NONE |
| 44 | M | 33 | n.d. | 1/29/16 | 3.85 | 786 | B/B | Experienced | FTC,RPV,TDF |
| 47 | F | 48 | n.d. | 1/13/14 | 4.53 | 36 | B/B | Experienced | ABC,LPV,RTV,RAL,3TC,AZT,EFV,TDF,NFV |
| 49 | M | 49 | n.d. | 1/30/14 | 4.50 | 872 | B/B | Experienced | AZT,SQV,LPV,RTV,ATV,3TC,d4T,TDF |
| 50 | M | 42 | n.d. | 8/27/14 | 4.74 | 51 | B/B | Experienced | FTC,ETR,TDF,RAL,LPV,RTV,AZT,ATV,ABC,3TC,NVP,EFV |
| 51 | M | 22 | n.d. | 8/27/14 | 5.43 | 507 | AG/AG | Naïve | NONE |
| 53 | M | 43 | n.d. | 8/18/14 | 4.82 | 282 | C/C | Experienced | ABC,3TC,RTV,ATV,AZT,APV |
| 54 | M | 42 | n.d. | 2/11/16 | 4.59 | 473 | B/B | Naïve | NONE |
| 55 | M | 44 | n.d. | 2/12/16 | 5.76 | 46 | G/A1 | Experienced | TDF,FTC,NVP,3TC,EFV,LPV,RTV |
| 56 | M | 51 | n.d. | 2/12/16 | 5.25 | 646 | B/B | Naïve | NONE |
| 57 | F | 50 | n.d. | 2/15/16 | 4.79 | 68 | B/B | Experienced | ABC,3TC,RPV,RTV,ATV,LPV,RAL,AZT,EFV,NFV,TDF |
| 58 | F | 23 | MTCT | 2/15/16 | 4.89 | 28 | B/B | Experienced | TDF,FTC,DRV,RTV,ddI,LPV,3TC,d4T,AZT |
| 59 | M | 27 | MSM | 2/16/16 | 4.08 | 552 | B/B | Naïve | NONE |
| 60 | M | 34 | n.d. | 2/17/16 | 4.83 | 317 | F1/F1 | Experienced | FTC,TDF,EFV |
| 61 | M | 46 | n.d. | 2/17/16 | 4.68 | 275 | C/B | Naïve | NONE |
| 63 | F | 41 | n.d. | 11/28/14 | 5.20 | 482 | C/C | Experienced | 3TC,AZT,LPV,RTV |
| 64 | F | 46 | n.d. | 11/17/14 | 4.22 | 428 | B/B | Experienced | 3TC,AZT,LPV,RTV |
| 65 | F | 54 | n.d. | 11/14/14 | 5.00 | 247 | B/B | Experienced | ABC,3TC,DRV,RTV,ATV,TDF,FTC,LPV |
| 66 | F | 45 | n.d. | 10/14/14 | 4.86 | 26 | B/B | Experienced | ETR,TDF,RAL,ABC,ddI,D4T,3TC,NVP,SQV,RTV,NFV,ATV |
| 67 | F | 22 | n.d. | 10/8/14 | 4.19 | 91 | B/B | Experienced | TDF,FTC,DRV,RTV,ddI,LPV,3TC,d4T,AZT |
| 68 | F | 30 | n.d. | 10/7/14 | 4.37 | 738 | A1/A1 | Naïve | NONE |
| 72 | M | 52 | n.d. | 3/18/16 | 4.84 | 157 | B/B | Experienced | FTC,TDF,EVG,COBI,RTV,DRV |

^a^ M, male; F, female. ^b^ Age at the time of sampling. ^c^ Most likely mode of HIV-1 transmission: HET, heterosexual; MSM, men who have sex with men; IVDU, intravenous drug user; MTCT, mother-to-child transmission; n.d., not determined. ^d^ date the blood sample was collected. ^e^ HIV-1 RNA plasma load (log_10_ copies/ml) at the time the blood sample was obtained. ^f^ CD4^+^ T-cell count (cells/mm^3^) at the time the blood sample was obtained. ^g^ HIV-1 subtype determined using the PR/RT- (*pol*) or the V3-coding region sequences with DEEPGEN™HIV proprietary pipeline and Geno2Pheno tools (<http://www.geno2pheno.org)>. ^h^ Patients treated (experienced) or not (naïve) with combination antiretroviral therapy (cART) at the time the blood sample was obtained. ^i^ Antiretroviral drugs used in each patient: AZT, zidovudine; ddI, didanosine; d4T, stavudine; 3TC, lamivudine; ABC, abacavir; TDF, tenofovir; FTC, emtricitabine; NVP, nevirapine; DLV, delavirdine; EFV, efavirenz; ETR, etravirine; RPV, rilpivirine; SQV, saquinavir; RTV, ritonavir; IDV, indinavir; NFV, nelfinavir; APV, amprenavir; LPV, lopinavir; ATV, atazanavir; TPV, tipranavir; DRV, darunavir; RAL, raltegravir; EVG, elvitegravir; DTG, dolutegravir; MVC, maraviroc; and COBI, cobicistat.

.
